# Combining lapatinib and pertuzumab to overcome lapatinib resistance due to NRG1-mediated signalling in HER2-amplified breast cancer

**DOI:** 10.18632/oncotarget.3296

**Published:** 2015-01-21

**Authors:** Wing-yin Leung, Ioannis Roxanis, Helen Sheldon, Francesca M. Buffa, Ji-Liang Li, Adrian L. Harris, Anthony Kong

**Affiliations:** ^1^ Department of Oncology, Molecular Oncology Laboratories, The Weatherall Institute of Molecular Medicine, University of Oxford, United Kingdom; ^2^ Department of Cellular Pathology, Oxford University Hospitals and Oxford Biomedical Research Centre, Oxford, United Kingdom; ^3^ New address: School of Cancer Sciences, University of Birmingham, Birmingham, United Kingdom

**Keywords:** lapatinib, pertuzumab, resistance, NRG1, HER2

## Abstract

Acquired resistance to lapatinib, an inhibitor of EGFR and HER2 kinases, is common. We found that reactivation of EGFR, HER2 and HER3 occurred within 24 hours of lapatinib treatment after their initial dephosphorylation. This was associated with increased expression of NRG1 in cells treated with lapatinib. Exogenous NRG1 partially rescued breast cancer cells from growth inhibition by lapatinib. In addition, both parental and lapatinib-resistant breast cancer cells were sensitive to SGP1, which inhibits binding of NRG1 and other HER3 ligands. Addition of pertuzumab to lapatinib further inhibited NRG1-induced signalling, which was not fully inhibited by either drug alone. In animal model, a combination of pertuzumab to lapatinib induced a greater tumor regression than either lapatinib or pertuzumab monotherapy. This novel combination treatment may provide a promising strategy in clinical HER2-targeted therapy and may inhibit a subset of lapatinib-resistant breast cancer, although the group of patients that will respond to this therapy requires further stratification.

## INTRODUCTION

The human epidermal growth factor receptor (HER, also known as ErbB) family consists of four transmembrane receptor tyrosine kinases, EGFR, HER2, HER3 and HER4 [1]. Dysregulation of the HER signalling pathway has been implicated in various epithelial cancers; for example, breast cancer, head and neck, and lung cancers [2]. Overexpression of HER2 protein and/or amplification of the HER2 gene occurs in around 20% of breast cancer patients and correlates with adverse prognosis and poor clinical outcome [3, 4]. HER2 overexpression correlates with tumour size, lymph node positivity, high tumour grade and aneuploidy [5]. There are two main types of HER2-targeted therapy: monoclonal antibodies (mAbs) and tyrosine kinase inhibitors (TKIs) [3]. Trastuzumab, pertuzumab (mAbs), trastuzumab emtansine (T-DM1, an antibody-drug conjugate) and lapatinib (TKI) were approved by the U.S. Food and Drug Administration and the European Medicines Agency and are now used in HER2-positive breast cancer patients. Trastuzumab is also used in HER2-positive gastric patients.

Pertuzumab is a humanised mAb that binds to the dimerisation domain (subdomain II) of the extracellular region of HER2, preventing its dimerisation with other HER receptors and thus inhibiting the HER signalling pathway [6, 7]. In preclinical studies, pertuzumab was found to inhibit NRG1-induced growth [8] and morphogenesis *in vitro,* and trigger rapid tumour regression in NRG1-dependent xenograft model [9]. The combination of pertuzumab and trastuzumab was shown to be synergistic in inhibiting the survival of HER2-overexpressing breast cancer cell line BT-474 [10]. In conjunction with chemotherapy, this combination has been shown to prolong PFS compared to trastuzumab in metastatic breast cancer as first line treatment [11] and improve pathological complete response (pCR) when used as a neoadjuvant therapy in early breast cancer [12]. Therefore, the FDA has approved the use of the combination of pertuzumab and trastuzumab with chemotherapy in metastatic breast cancer and early HER2-overexpressing breast cancer in the neoadjuvant setting.

Lapatinib is a small molecule chemical inhibitor designed to target the tyrosine kinase domain (TKD) of EGFR and HER2 [13, 14]. Lapatinib reversibly binds to the ATP-binding pocket of the TKD and prevents phosphorylation [14]. It has been proven successful in inhibiting the phosphorylation of HER receptors and its downstream targets such as Akt and MAPK [14, 15], and slowing down tumour growth *in vitro* and *in vivo* [13, 14]. Lapatinib is used in patients with advanced HER2-positive breast cancer that have progressed after previous trastuzumab containing regimens and its efficacy as a single targeted therapy with chemotherapy has been shown [16, 17]. Lapatinib in combination with trastuzumab and chemotherapy has been reviewed in neoadjuvant studies such as CHER-LOB [18], NSABP B-41 [19] and neoALTTO trials [20]. Recently, a meta-analysis of six randomised trials had concluded that lapatinib in combination with chemotherapy achieved a lower pCR and higher risk of toxicity compared to trastuzumab with chemotherapy in the neoadjuvant setting [21]. Furthermore, a phase III study reported in the 2012 American Society of Clinical Oncology Annual Meeting found that, metastatic HER2-positive breast cancer patients who received lapatinib and chemotherapy had shorter PFS than those receiving trastuzumab and chemotherapy [22].

Several models have been proposed to explain the resistance to lapatinib in HER2-overexpressing breast cancer. Lapatinib derepresses FOXO3a, which stimulates oestrogen receptor (ER) transcription and leads to the co-dependence on ER and HER2 signalling [23]. Lapatinib activates ER-induced overexpression of AXL, which stimulates downstream PI3K/Akt pathway and promotes survival [24]. Derepression of FOXO3a also upregulates HER3 [25]. Lapatinib triggers a calcium stress response that activates RelA, which inhibits apoptosis [26]. Recently, Xia *et al.* reported that NRG1 autocrine stimulation of EGFR/HER3 signalling mediates acquired lapatinib resistance [27], although the exact mechanism was not elucidated.

Here, we report that NRG1 stimulation activated HER3 and HER4 signalling despite the inhibition incurred by lapatinib. However, a novel combination of HER2-targeted therapies, lapatinib and pertuzumab, inhibited these NRG1-stimulated HER signalling pathways. The anti-tumour activity of this drug combination was verified *in vitro* and *in vivo* and was confirmed to be more efficient than either drug alone.

## RESULTS

### Lapatinib could not sustain the inhibition of HER receptor signalling and upregulates NRG1 expression

**Figure 1:**
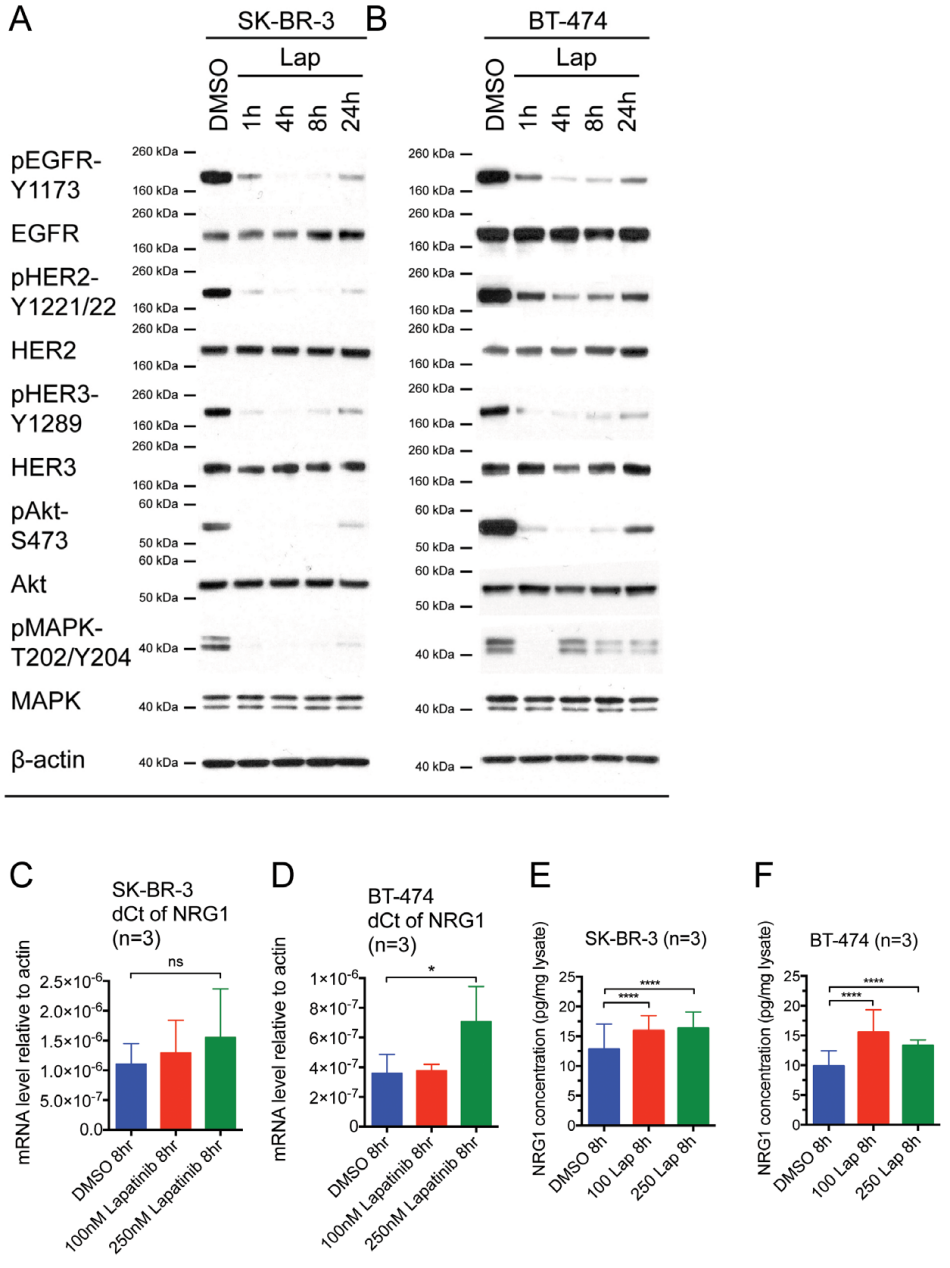
Reactivation of HER receptors occurs within 24 hours of lapatinib treatment, correlating with an increase in NRG1 expression (A) SK-BR-3 and (B) BT-474 cells were treated with 100 nM lapatinib for the indicated times or DMSO solvent control before immunoblotting for the indicated proteins. β-actin blot served as loading control in all experiments. Reproducible representative results of at least three independent experiments are shown. (C) SK-BR-3 and (D) BT-474 cells were treated with the indicated doses of lapatinib or DMSO control for 8 hours. The mRNA expression of NRG1 was assessed by qPCR and the relative expression level of NRG1 with respect to β-actin control was calculated using the dCt method. Three independent experiments were performed in three technical replicates each time. (E) SK-BR-3 and (F) BT-474 cells were treated with the indicated concentrations of lapatinib or DMSO control for 8 hours. Equal amount of complete cell lysates were applied to NRG1 ELISA kit according to manufacturer protocol. Three independent experiments were performed with two technical replicates for ELISA. (Error = SD; *p<0.05, **p<0.01, ****p<0.0001).

To assess the acute response that may counteract lapatinib inhibition, we treated two commonly used HER2-overexpressing breast cancer cell lines, SK-BR-3 and BT-474, with lapatinib for 24 hours. After 4 hours of lapatinib treatment, the phosphorylation of EGFR, HER2 and HER3 in both cell lines was reduced. However, these receptors started to rephosphorylate after 8 to 24 hours whilst on treatment (Fig. 1A and B). In SK-BR-3 cells, the phosphorylation of downstream effectors of the HER signalling pathway, MAPK and Akt, were inhibited by lapatinib after 1 hour, but started to recover after 24 hours of lapatinib treatment (Fig. 1A). In BT-474 cells, the dephosphorylation and reactivation of Akt correlated with its upstream HER3 receptor in a similar way to SK-BR-3, but it was more robust and rapid in BT-474. However, phospho-MAPK followed a different pattern (Fig. 1B). The phosphorylation of MAPK decreased to an undetectable level after 1 hour of treatment; however, it rose to near basal level at 4 hours and decreased again from 8 to 24 hours.

Xia et al. (2013) reported a model of acquired lapatinib resistance, in which EGFR/HER3 signalling was induced by NRG1 autocrine stimulation [27]. However, the short-term effect of lapatinib on NRG1 expression was not shown. We hypothesised that the reactivation of EGFR, HER2 and HER3 as well as the downstream pathways during the first 24 hours of lapatinib treatment could be mediated by NRG1. We showed that lapatinib treatment in 100 nM and 250 nM for 8 hours induced an increasing trend of NRG1 mRNA expression in SK-BR-3 cells (Fig. 1C), although the increase was not statistically significant. In BT-474 cells, NRG1 mRNA level increased significantly after the cells were treated with 250 nM lapatinib for 8 hours (p<0.05) but not with 100 nM lapatinib (Fig. 1D). We further confirmed that there was a small but statistically significant increase in NRG1 protein expression upon lapatinib treatment using ELISA. In SK-BR-3 and BT-474 cells, both 100 nM and 250 nM lapatinib increased NRG1 expression in the cell lysates (Fig. 1E and F, all p <0.0001 compared to DMSO). However, NRG1 level in the medium was too low to be detected by ELISA (data not shown).

### Acquired lapatinib resistance is mediated by HER3 ligands

**Figure 2:**
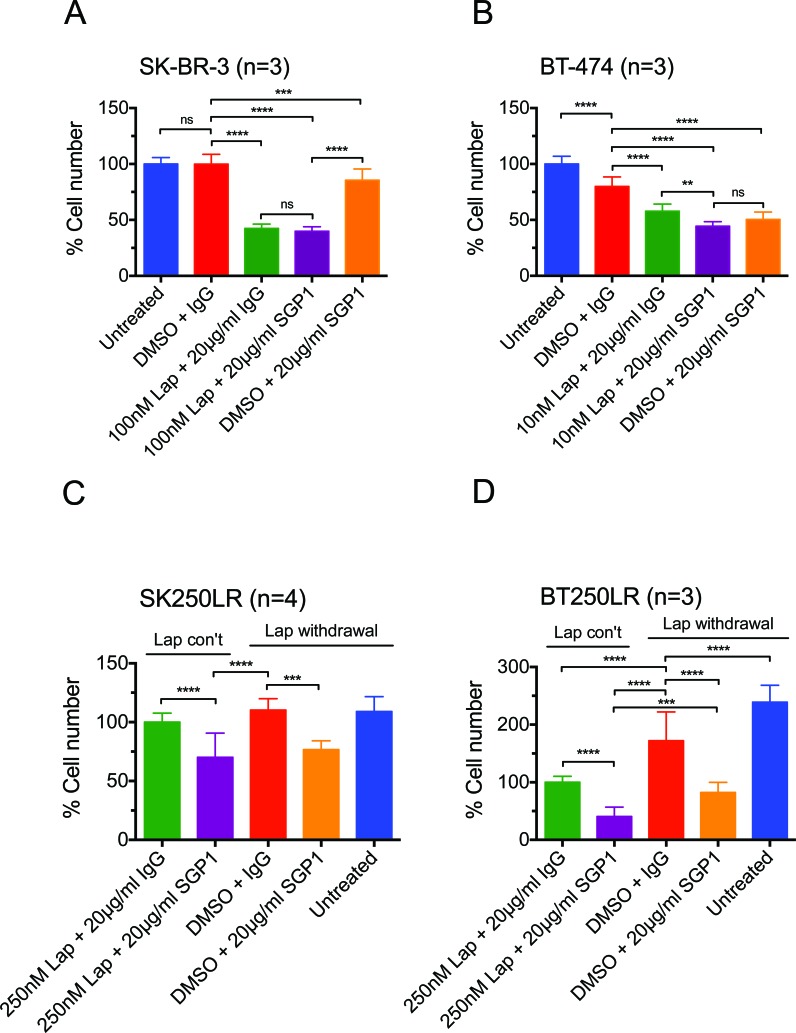
The inhibitory effect of lapatinib and/or SGP1 on cell growth in parental and lapatinib-resistant HER2-over-expressing breast cancer cells (A) SK-BR-3 and (B) BT-474 cells were treated with the indicated agents for 3 days. (C) SK250LR and (D) BT250LR cells were seeded and maintained in continuous lapatinib treatment the night before the experiment. On the next day, the drug was taken off and the indicated agents were applied for 3 three days. The cells were then trypsinised and counted. The percentage cell number was normalised to (A and B) the untreated control or (C and D) continuous 250nM lapatinib plus 20 μg/ml non-specific IgG control. At least three independent experiments were done with three technical replicates. (Error = SD; **p<0.01, ***p<0.001, ****p<0.0001).

Since NRG1 level was shown to be increased during the first 8 hours of lapatinib treatment (Fig. 1E and F) and it has also been shown to mediate acquired lapatinib resistance [27], we assessed the effect of HER3 monoclonal antibody, SGP1, that could block HER3 ligands from binding the receptors [28] in both parental and lapatinib-resistant SK-BR-3 and BT-474 cells. SGP1 alone or lapatinib alone reduced the cell number of the parental SK-BR-3 and BT-474 cells significantly (both p<0.001 compared to the control) (Fig. 2A and B). The addition of SGP1 to lapatinib was more effective than lapatinib alone in the parental BT-474 cells and not SK-BR-3 cells, although this was not more effective than SGP1 alone in BT-474 cells (Fig. 2A and B). We generated lapatinib-resistant cell lines from HER2-overexpressing breast cancer cell lines SK-BR-3 and BT-474 by chronic lapatinib treatment up to the concentration of 250 nM over 10 months. The resistant cell lines had reduced sensitivity to lapatinib treatment (Suppl. Fig. 1). In the resistant cells, there was an increase of cell number when lapatinib was withdrawn in BT250LR cells (Fig. 2D) but not in SK250LR cells (Fig. 2C). SGP1 with or without lapatinib withdrawal could reduce the cell number significantly in both resistant cells (Fig. 2C and D). Thus, we showed that both parental and resistant SK-BR-3 and BT474 cells were sensitive to SGP1, which inhibits binding of NRG1 and other HER3 ligands.

### Exogenous NRG1 stimulation induces acquired resistance to lapatinib

To further confirm the role of NRG1 in mediating resistance to lapatinib, we stimulated SK-BR-3 and BT-474 cell with exogenous NRG1, while they were concurrently treated with lapatinib. In both cell lines, lapatinib decreased the cell number significantly (p<0.0001 compared to DMSO in both cell lines), but the addition of NRG1 to lapatinib treatment recovered the cell number to the control level (p<0.0001 compared to lapatinib alone in both cell lines) (Suppl. Fig. 2A and B). The above results provided evidence that NRG1, a HER3 ligand, rescues HER2-overexpressing cell lines SK-BR-3 and BT-474 cells from the inhibitory effect of lapatinib.

### Combination of lapatinib and pertuzumab inhibits NRG1-induced signalling

Although NRG1 autocrine signalling was proposed to be one of the underlying mechanisms of lapatinib resistance, no effective treatment option has been proposed [27]. NRG1 is a ligand for HER3 and HER4, and the release of NRG1 and other HER ligands could induce HER2 dimerisation with other HER receptors [1]. This is supported by a report that lapatinib could increase HER2/HER3 and EGFR/HER2 dimerisation [29]. Thus, we hypothesised that the HER2 dimerisation inhibitor, pertuzumab, could inhibit NRG1-mediated HER receptor signalling induced by lapatinib treatment [30].

**Figure 3:**
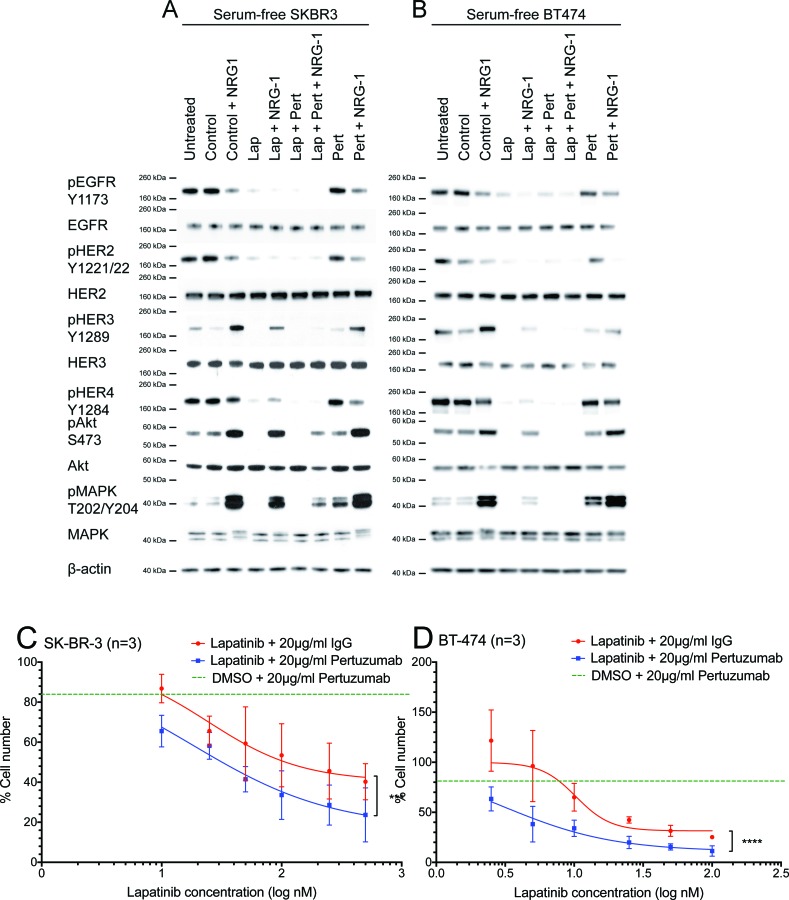
The effect of the lapatinib/pertuzumab combination on NRG-1 stimulated HER signalling, cell growth and viability (A) SK-BR-3 and (B) BT-474 cells were grown in serum-free culture media for 24 hours before being treated with 100 nM lapatinib and/or 20 μg/ml pertuzumab for 1 hour. DMSO and/or 20 μg/ml non-specific IgG were used as treatment controls. In the last 10 minutes of treatment, 10 ng/ml NRG1 was added as indicated before immunoblotting for the indicated proteins. β-actin blot served as loading control in all experiments. Reproducible representative results of at least three independent experiments are shown. None of the two commercially available anti-HER4 antibodies produced convincing bands in western blot (data not shown). (C) SK-BR-3 and (D) BT-474 cells were treated with the indicated doses of lapatinib or DMSO control, and 20 μg/ml pertuzumab or non-specific human IgG control before the cells were trypsinised and counted. Cell number were normalised with DMSO and IgG control. Three independent experiments were done with three technical replicates. Three independent experiments were performed with three technical replicates. (Error = SD; ***p<0.001, ****p<0.0001).

In serum-free media, lapatinib inhibited phosphorylation of all HER receptors and the downstream Akt and MAPK in both parental cell lines (Fig. 3A and B). When the cells were stimulated by NRG1, lapatinib could still inhibit phosphorylation of EGFR and HER2; however, there was an increase in phosphorylation of HER3, HER4, MAPK and Akt compared to lapatinib alone. These results suggested that NRG1 stimulation could partially reverse the inhibition induced by lapatinib. We showed that pertuzumab alone did not inhibit any HER receptors nor downstream targets analysed in both SK-BR-3 and BT-474 cells; however, in the presence of NRG1 stimulation, pertuzumab inhibited phospho-EGFR, phospho-HER2 and phospho-HER4 (Fig. 3A and B). This result indicated that pertuzumab inhibited as well as decreased NRG1-induced HER3 activation ligand-induced HER signalling in these cells.

When the cells were stimulated by NRG1, the combination of lapatinib and pertuzumab inhibited phospho-HER3, phospho-HER4, phospho-Akt and phospho-MAPK more than lapatinib alone. Both phospho-HER3 and phospho-HER4 were inhibited to levels comparable to no NRG1 stimulation in SK-BR-3 cells (Fig. 3A). Phospho-Akt and phospho-MAPK in SK-BR-3 cells were still higher than basal levels when NRG1-stimulated cells were treated with the combination (Fig. 3A); however, in BT-474 cells, both downstream effectors were completely inhibited (Fig. 3B). This experiment showed that the combination of lapatinib and pertuzumab could inhibit NRG1-stimulated HER signalling pathways, which were not completely inhibited by either drug alone.

### Effect of the combination of lapatinib and pertuzumab *in vitro*

The additive effect of the combination of lapatinib and pertuzumab on the inhibition of NRG1-stimulated HER signalling suggested that this combination might have improved anti-tumour activity. In total cell counting experiments, the combination of lapatinib and pertuzumab (blue line) was shown to reduce total cell number more than lapatinib alone at any concentration (red line) and pertuzumab alone (green dashed line) in both SK-BR-3 and BT-474 cells (Fig. 3C and D). The difference between the combination and lapatinib alone was significant in both SK-BR-3 (p<0.001) and BT-474 (p<0.0001) cells, suggesting the combination effect of the two drugs may be additive.

The additive effect of the drug combination was further confirmed by an apoptotic assay. Although the combination of lapatinib and pertuzumab induced the highest percentage of apoptotic cells in both cell lines, the change was only statistically significant compared to lapatinib alone (+IgG control) in BT474 but not SK-BR-3 cells (Suppl. Fig. 3A and B and Suppl. Fig. 4). However, the combination was significantly more effective than pertuzumab alone in both cell lines. In order to further understand the differences between the two cell lines, the effect of the drug combination was investigated in the lapatinib-resistant cell lines as below.

### Effect of the combination of lapatinib and pertuzumab in lapatinib-resistant cell lines

**Figure 4:**
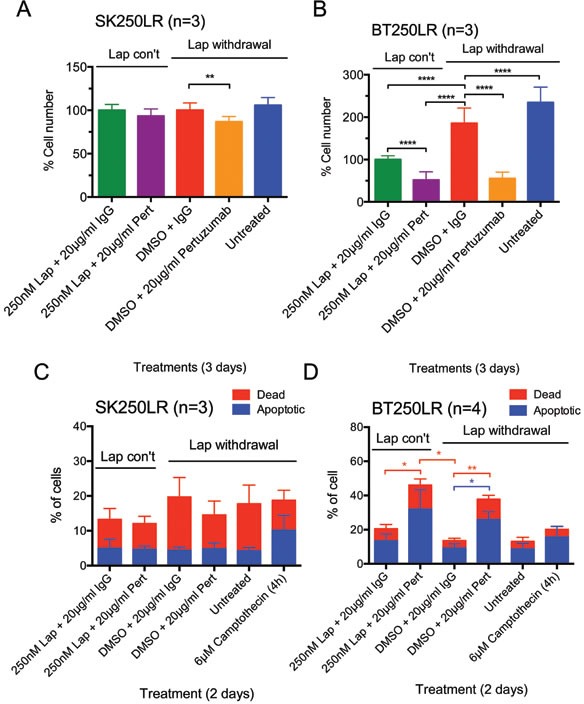
Effect of pertuzumab with or without continuous lapatinib treatment in lapatinib-resistant cell lines (A) SK250LR and (B) BT250LR cells were seeded and maintained in continuous lapatinib treatment the night before the experiment. On the next day, the drug was taken off before being treated with the indicated treatments for 3 days. Cells were then trypsinised and counted. The percentage cell number was normalised with continuous 250nM lapatinib/non-specific IgG. Three independent experiments with three technical replicates were performed. (C) SK250LR and (D) BT250LR cells were treated with the indicated treatment for 2 days. Cells were then trypsinised, collected and stained with Annexin V-Alexa Fluor 647 and propidium iodide. Stained cells were analysed using CyAn FACS analyser. Three independent experiments were performed. (Error = SD; *p<0.05, **p<0.01, ****p<0.0001).

The drug combination was tested on lapatinib-resistant cell lines SK250LR and BT250LR to understand whether this could overcome acquired lapatinib resistance. Pertuzumab slightly reduced cell number (p<0.01) compared to the DMSO (+ IgG control) only when lapatinib was withdrawn in SK250LR cells but there was no statistically significant difference between all other groups (Fig. 4A), indicating that pertuzumab was not very effective in SK250LR cells. This is consistent with the apoptotic assay, which showed that pertuzumab did not cause any change in the percentage of apoptotic cells with or without lapatinib in SK250LR cells (Fig. 4C and Suppl. Fig. 5A to F). In BT250LR cells, withdrawal of lapatinib resulted in a statistically significant higher number of cell count compared to those with continuous lapatinib treatment (p<0.0001, Fig. 4B). Pertuzumab significantly reduced cell number of BT250LR whether or not lapatinib was present (both p<0.0001), which is consistent with the apoptotic assay (Fig. 4D, Suppl. Fig. 5G to L). These experiments showed that lapatinib-resistant cell line BT250LR was sensitive to pertuzumab with or without continuous lapatinib treatment, but not SK250LR.

### Lapatinib-resistant SK-BR-3 lost its dependence on HER2 signalling pathway

**Figure 5:**
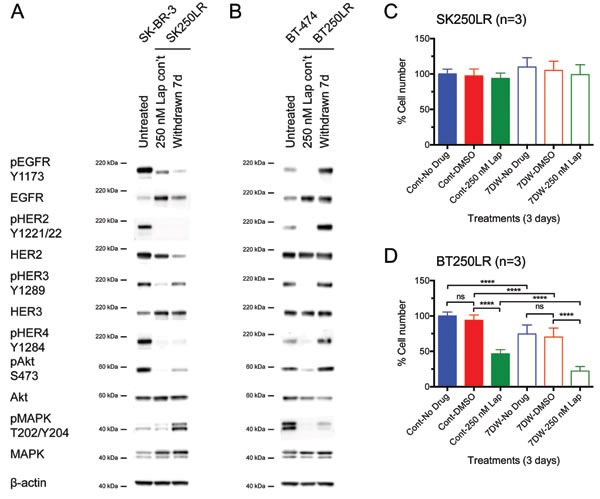
Effect of lapatinib withdrawal from lapatinib-resistant cell lines on their HER signalling profile and lapatinib sensitivity (A) SK250LR and (B) BT250LR cells were continuously treated with 250 nM lapatinib or had the drug withdrawn for 7 days before being immunoblotted for the indicated proteins. Reproducible representative results of at least three independent experiments are shown. (C) SK250LR and (D) BT250LR were either continuously treated with 250 nM lapatinib treatment (solid bars, Cont) or had withdrawal of the drug (hollow bars, 7DW) for 7 days before they were re-seeded to 24-well plates. Cells were then treated with DMSO or 250 nM lapatinib for 3 days, and then trypsinised and counted. The percentage of cell number was normalised to the cells treated with lapatinib continuously and then no drug for 3 days (Cont-No drug). Three independent experiments were performed with three technical replicates. (Error = SD; ****p<0.0001).

In previous experiments, the resistant cells were continuously treated with lapatinib until being seeded for cell counting experiments. To investigate the reasons for SK250LR cell line not responding to the combined anti-HER2-targeted therapy, lapatinib was removed from the lapatinib-resistant cell lines for 7 days (7D) before further western blot and cell counting experiments. The HER signalling profile of these 7D-lapatinib-withdrawn cells was compared to cells that were continuously treated with lapatinib and untreated parental cells. In SK250LR cells, phospho-EGFR, phospho-HER2 and phospho-HER4 did not reactivate after lapatinib was removed for 7 days (Fig. 5A). Furthermore, the expression levels of EGFR and HER2 decreased after 7D-lapatinib withdrawal, indicating that the cells might have developed permanent changes in the cell biology, in which they were no longer dependent on EGFR and HER2 signalling. In fact, the cell counting experiments revealed that SK250LR cells were still insensitive to lapatinib even after 7 days of lapatinib withdrawal (Fig. 5C). HER3 and Akt were slightly rephosphorylated (Fig. 5A). They appeared to correlate with each other, as Akt is the main downstream effector of HER3. Phospho-MAPK significantly increased in SK250LR cells after 7D-lapatinib withdrawal; however, this change was not correlated to the phosphorylation any of the HER receptors.

In BT250LR cells, all the HER receptors and downstream proteins rephosphorylated after lapatinib was removed for 7 days (Fig. 5B). The levels of phospho-EGFR, phospho-HER2, phospho-HER4 and phospho-Akt in BT250LR after 7-day withdrawal were even higher than those in untreated BT-474 cells. Phospho-HER3 recovered to similar level of untreated BT-474 cells, but phospho-MAPK was still below the basal level. There was no difference in the expression levels of EGFR, HER2 and HER3 before and after 7D-lapatinib withdrawal. The reintroduction of 250 nM lapatinib after 7 days of withdrawal (7DW-250 nM Lap) caused a decrease in the cell number of BT250LR cells-7DW compared to DMSO control (p<0.0001), which was more than those which were continuously treated with lapatinib (Cont-250 nM Lap) (Fig. 5D). This indicates that the cells were only transiently adapted to lapatinib, and removing lapatinib from BT250LR cell line reverted its resistance resulting in BT250LR-7DW becoming more sensitive to lapatinib.

By comparing the differences in HER signalling profile between SK250LR and BT250LR cell lines, it is suggested that BT250LR cells responded to pertuzumab treatment because they were still dependent on the HER2 signalling, whilst SK250LR cells had downregulation of HER2 and decreased pHER2 levels, suggesting a shift of dependency from HER2 signalling and hence being less sensitive to pertuzumab.

### Effect of the combination of lapatinib and pertuzumab *in vivo*

**Figure 6:**
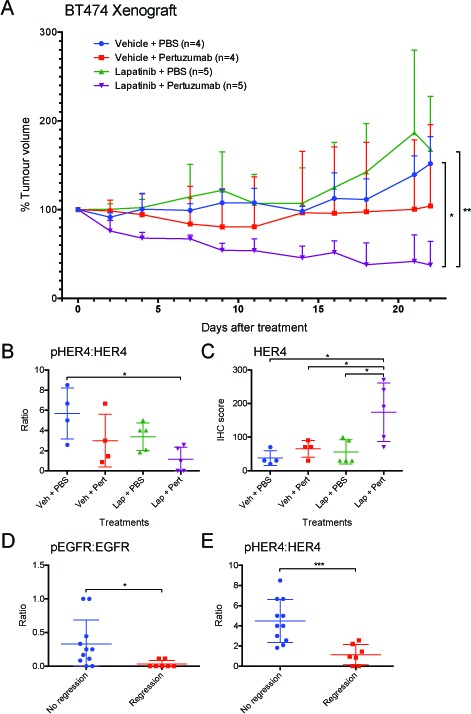
Anti-tumour effect of lapatinib/pertuzumab combination *in vivo* (A) Mice bearing BT-474 xenograft were treated with the indicated treatments. (Lapatinib: 50 mg/kg five times per week; pertuzumab 12 mg/kg loading dose followed by 6 mg/kg maintenance dose per week). Tumour volume was measured three times a week (as indicated at each data point) for 22 days. The tumour volume was normalised to the tumour volume on day 0. (B-E) Indicated HER receptors and their phosphorylated forms (shown in the ratio of phosphorylated:total receptor) were scored in terms of IHC staining intensity and percentage of cells. The scores were then grouped according to (B and C) the treatments that the mice received or (D and E) tumour regression status. (Error = SD; *p<0.05, **p<0.01, ***p<0.001).

The anti-tumour efficacy of the drug combination was further tested in a BT-474 mouse xenograft model. In the clinical setting, a lower dose of lapatinib has been used when combined with trastuzumab (+/− chemotherapy) due to possible additional toxicity and expected increased efficacy. For example, in the neoALTTO trial, a 1500 mg lapatinib dose was used for the lapatinib group but 1000 mg was used when combined with trastuzumab [20]. To demonstrate the additive effect of the drug combination, we also used a lower dose of lapatinib, which did not reduce tumour size in this experiment (Fig. 6A). The results in the pertuzumab treated group varied, with two tumour regressed but another two progressed. All tumours in the combination treatment group regressed and this change was significantly different from the vehicle group (p<0.05) and the lapatinib group (p<0.01). The above results suggested that the combination of lapatinib and pertuzumab outperformed either monotherapy *in vivo*.

Immunohistochemistry staining was performed to detect all the HER receptors and their phosphorylated forms as well as the expression of Ki-67 and cleaved caspase-3 in the xenografted tumours. The tumours that received combination treatment expressed significantly higher levels of HER4 than all other treatment groups (p <0.05) (Fig. 6C). There was no significant difference in phospho-HER4 among all treatment groups (not shown); however, since the combination treatment group had high HER4 expression, the ratio of pHER4:HER4 in the combination group was significantly lower than that of the vehicle control group (p<0.05) (Fig. 6B). Although trastuzumab has been shown to downregulate HER2 [31], there was no decrease of HER2 level by lapatinib or pertuzumab or their combination *in vivo* (Suppl. Fig. 6). There were also no significant differences in other protein markers between the different groups (Suppl. Fig. 6).

The results of IHC staining were also compared with the regression status of the tumours. The regressed tumours had significantly reduced ratio of pEGFR:EGFR (p<0.05) (Fig. 6D) and pHER4:HER4 (p<0.001) (Fig. 6E) compared to the tumours without regression, suggesting the inhibition on EGFR and HER4 might be the cause of tumour regression. There was also higher expression of HER4 in the regressed tumours (p<0.05), but there were no significant differences between regressed and non-regressed tumours in other proteins (Suppl. Fig. 7).

## DISCUSSION

NRG1 has been implicated in lapatinib resistance [27] as well as overcoming the inhibitory effect of gefitinib [32] and trastuzumab [33] treatment in HER2-overexpressing breast cancer cells. Our study demonstrated that the NRG1 protein expression levels increased in response to lapatinib treatment in HER2-overexpressing breast cancer cell lines, and lapatinib could not sustain its inhibition of HER receptor signalling. The use of SPG1 antibody suggested that HER3 ligands may contribute to lapatinib resistance. Moreover, we showed that pertuzumab could enhance response to lapatinib in BT-474 cells and xenograft models.

A very recent study revealed that high NRG1 expression predicts a decreased recurrence-free survival in HER2-overexpressing breast cancer patients compared to patients with low NRG1 expression [27], suggesting the clinical importance of NRG1. Although their study aimed to demonstrate the role of NRG1 autocrine signalling in the acquired lapatinib resistance, they combined the data of all patients receiving chemotherapy, tamoxifen or systematically untreated; therefore, this analysis could not address the association between NRG1 expression and lapatinib response. To date, there is only one published clinical trial involving lapatinib (EGF104911) that measured NRG1 level in tumours by immunohistochemistry (IHC), but they did not mention any correlation between NRG1 and clinical benefits from lapatinib monotherapy [34]. We propose for further studies to be done to validate NRG1 as a predictive biomarker for lapatinib sensitivity. Since ADAM10 and 17 have been shown to play a role in mediating acquired resistance to trastuzumab through the shedding of HER ligands [33, 35], it may be important to assess the role of ADAM 10 and 17 proteases as well as other HER ligands in mediating lapatinib resistance in HER2-positive breast cancer.

The addition of pertuzumab to lapatinib inhibited the phosphorylation of HER3 and HER4, suggesting that HER3 and HER4 still preferentially bind to HER2 even in the presence of lapatinib. However, the phosphorylation of downstream MAPK and Akt was not completely inhibited. This may be caused by the residue kinase activity of the HER receptors, or the dimerisation of HER3 and HER4 with EGFR or each other. Thus, it may be important to assess the different dimerisation patterns in relation to lapatinib treatment and resistance [36]. Since neratinib could overcome trastuzumab resistance [37] and lapatinib resistance [27], it will be interesting to assess whether the combination of pertuzumab and neratinib will also be additive *in vitro* and *in vivo* for HER2-positive breast cancer.

HER4 expression levels were high in the tumours treated with the combination of lapatinib and pertuzumab, suggesting HER4 may mediate the effect of the drugs on tumour regression. HER4 has been implicated in both favourable and poor prognosis [38], but the cellular functions of HER4 vary among different isoforms as well as its localisation [39]. Our lab has previously shown that HER4 cleavage and nuclear translocation mediates acquired resistance to trastuzumab; and nuclear HER4 but not cytoplasmic HER4 is associated with poor outcome in HER2-positive breast cancer [40]. Therefore, further analysis of HER4 isoforms as well as its proteolytic cleavage and localisation may be required to understand the correlation of HER4 expression and lapatinib-induced tumour regression. There was no significant difference in the expression of Ki-67 and cleaved caspase-3 between the combination group and other different treatment groups in the BT-474 xenograft experiment, although the combination group was the most effective in inhibiting tumour growth. This could be because the analysed tumours are the residual tumour cells that were either innately resistant or had developed acquired resistance to the treatment(s). There is also a possibility that the tumour regression may not be caused by the difference in growth rate or apoptosis between the groups. The other potential mechanisms to consider include vessel growth inhibition, changes in vessel perfusion and ADCC which could be induced by pertuzumab [41].

Total cell counting and apoptotic assays showed that SK250LR cell line was not sensitive to the combination treatment of lapatinib and pertuzumab. The innate resistance to pertuzumab may be due to the permanent change in cell biology such that they are no longer dependent on EGFR and HER2 signalling. However, the BT250LR cell line was still dependent on HER2 signalling and responded to pertuzumab treatment. Our data shows that the combination of lapatinib and pertuzumab or pertuzumab alone may be effective in a subset of lapatinib-resistant cancers, which could be further confirmed *in vivo* by testing in a BT250LR xenograft model.

One limitation of our study was that it was carried out in only two cell lines, SK-BR-3 and BT-474, although the lapatinib-resistant variants of these 2 cell lines were included. These two cell lines appeared to have different mechanisms of acquired resistance to lapatinib. Therefore, more acquired lapatinib-resistant cell lines could be generated from other lapatinib-sensitive HER2-overexpressing breast cancer cell lines to further understand the difference mechanisms of acquired lapatinib resistance. A recent report showed that anti-HER3 monoclonal antibody was synergistic with lapatinib in PC9ZD, a gefitinib-resistant lung cancer cell line which harbours the T790M mutation [42]. As we have shown that the NRG1/HER3 pathway was one of the mechanisms of acquired lapatinib resistance in HER2-overexpressing breast cancer cell lines, this pathway might also have a potential role in breast cancer cells that have innate resistance to lapatinib. Thus, it would be important to assess the role of HER3 ligands and the effect of HER3 monoclonal antibodies in combination with lapatinib in a larger panel of lapatinib-sensitive and innate-resistant breast cancer cell lines.

It may also be important to consider other pathways which may affect HER receptor signalling and lapatinib sensitivity. For example, a recent report showed that neurotensin (NTS) and its high affinity receptor (NTSR1) enhances EGFR, HER2, and HER3 activation, but lapatinib could reduce the tumour growth of breast cancer cells overexpressing NTS and NTSR1 [43]. Therefore, it could be interesting to further assess the cross-talk between neurotensinergic and the HER pathways in relation to lapatinib sensitivity and resistance. It may also be useful to use a high-throughput combinatorial drug screening approach to identify compensatory pathways that mediate lapatinib resistance and to target them with other targeted agents in combination with lapatinib to overcome its resistance and to achieve durable clinical in order benefit [44].

Although lapatinib plus pertuzumab may be a useful combination strategy for anti-HER2 therapy, it will require further comparison with trastuzumab plus pertuzumab or trastuzumab plus lapatinib or the triple combination in preclinical models and in future human patient trials. Furthermore, since this study was initiated, the roadmap for anti-HER2 treatments has changed. For metastatic breast cancer, the combination of pertuzumab and trastuzumab with docetaxel chemotherapy is now recommended as the first-line treatment. T-DM1 is increasingly used as the second-line anti-HER2 treatment since EMILIA trial showed that T-DM1 was superior to lapatinib plus capecitabine in prolonging progression-free and overall survivals, and it caused less toxicity in HER2-positive breast cancer patients previously treated with trastuzumab and a taxane [45]. This means that lapatinib with capecitabine is increasingly being used as a third-line anti-HER2 treatment for metastatic breast cancer. Although we showed that pertuzumab and lapatinib could potentially be useful in the setting of lapatinib resistance, this is uncertain in the context of patients who have previously failed three lines of anti-HER2 treatments, namely pertuzumab and trastuzumab with docetaxel followed by T-DM1 and lapatinib with capecitabine. This will needs to be tested in preclinical models first. However, the combination may still be potentially useful for patients who have started on lapatinib containing regimen first and have not had pertuzumab.

In summary, we have found that lapatinib could not sustain the inhibition of HER receptor signalling. This is correlated with an increased upregulation of NRG1 expression in HER2-overexpressing cells. Both the parental and lapatinib-resistant SK-BR-3 and BT-474 cells were sensitive to SGP1, which inhibits the binding of NRG1 and other HER3 ligands. The exogenous NRG1 stimulation diminished the inhibitory effect of lapatinib in HER2-overexpressing cells. The addition of pertuzumab to lapatinib inhibited NRG1-stimulated HER3 and HER4 signalling in both SK-BR-3 and BT-474 cells, although the additive effect was only seen in the parental and resistant BT-474 cells as well as BT-474 xenograft models. This novel combination treatment may provide a promising strategy in clinical HER2-targeted therapy and may inhibit a subset of lapatinib-resistant breast cancer, although the group of patients that will respond to this therapy requires further stratification.

## MATERIALS AND METHODS

### Cell culture and reagents

Human breast cancer cell lines SK-BR-3 and BT-474 were obtained from cell services at Cancer Research UK (Lincoln's Inn Fields laboratory), which has a stringent quality control in cell authenticity and has incorporated short-tandem repeat (STR) profiling for cell line validation. Lapatinib-resistant cell lines SK250LR and BT250LR were generated by chronic exposure of increasing concentration of lapatinib from 25 nM, 50 nM, 100 nM to 250 nM for 10 months, then the cells were maintained in 250 nM lapatinib. Fresh lapatinib was replaced every 3 days. Lapatinib was purchased from Selleck Chemicals. Pertuzumab was generously provided by Roche. SGP1 antibody was obtained from Cancer Research Technology.

Recombinant NRG1 was obtained from Sigma-Aldrich. Human non-specific IgG control was purchased from R&D Systems.

### SDS-PAGE and Western blotting

The procedures of SDS-PAGE and Western blotting have been previously described [33, 35]. Primary antibodies to EGFR (1:5000), phospho-HER2 (Y1221/22) (1:1000), HER2 (1:10000), phospho-HER3 (Y1289) (1:1000), HER3 (1:10000), phospho-Akt1/2 (S473) (1:1000), Akt1/2 (1:5000), phospho-p42/44-MAPK (T202/Y204) (1:1000), p42/44-MAPK (1:5000) and β-actin (1:2500) were from Cell Signalling Technology. Antibodies recognising phospho-EGFR (Y1173) (1:1000) and phospho-HER4 (Y1056) (1:1000) were obtained from Santa Cruz Biotechnology. Anti-HER3 (1:10000) antibody was purchased from Abcam. HRP-conjugated anti-rabbit IgG secondary antibody (1:10000) was purchased from Life Technologies. After antibody incubation, the membrane was visualised using ImageQuant LAS 4000 mini system (GE healthcare).

### ELISA

ELISA detection of NRG1 was performed using the Human NRG1-β1/HRG1-β1 DuoSet kit (R&D Systems) and the procedures were done according to manufacturer's protocol. A six-point standard curve was produced using a 2-fold serial dilution of a standard of 4000 pg/ml NRG1 and plotted using a fourth order polynomial function in GraphPad Prism 6. The concentration of NRG1 in each sample was calculated using the standard curve and normalised with the protein concentration as a surrogate for cell number.

### RNA silencing

AllStars Negative Control (Qiagen) was used as scramble siRNA. siNRG1-9 (5′-UUUCAAACCCCUCGAGAUA-3′) and siNRG1-11 (5′-GGGGAGUGCUUCAUGGUGA-3′) were designed by Thermo Scientific and synthesised by Life Technologies. siRNA was diluted in Opti-MEM medium to the final concentration as indicated in the experiments for each reaction. DharmaFECT 1 Transfection Reagent was diluted in Opti-MEM medium (5 μl reagent in 15 μl medium for each reaction) and incubated at RT for 5 minutes. Diluted siRNA was mixed with the DharmaFECT 1 solution and then incubated at RT for another 20 minutes. Cells maintained in antibiotics-free medium supplemented with 10% FBS were seeded on a 6-well plate with the DharmaFECT 1-siRNA complexes. The culture medium was replaced 24 hours post-transfection.

### Quantitative PCR

RNA was isolated from cells using RNeasy mini kit (Qiagen) according to manufacturer's protocol. cDNA was synthesised using the High-Capacity cDNA Reverse Transcription kit (Life technologies). Quantitative PCR were performed using the following primers, together with SensiMix SYBR Hi-ROX kit (Bioline): NRG1, 5′-GCTTCATGGTGAAAGACCTTTCA-3′ and 5′-ATTACGTAGTTTTGGCAGCGATC-3′; β-actin, 5′-ATTGGCAATGAGCGGTTC-3′ and 5′-GGATGCCACAGGACTCCAT-3′. Triplicate of the cDNA sample were analysed using Applied Biosystems 7900HT Fast Real-Time PCR System.

### Real-time live cell imaging

Approximately 10,000 cells per well (for SK-BR-3 and SK250LR) or 20,000 cells per well (for BT-474 and BT250LR) were seeded in triplicate on 24-well tissue culture plates (Essen BioScience), allowed to adhere and enter log phase overnight. Each triplicate was treated with the indicated agents. Cell proliferation was monitored by IncuCyte Kinetic Live Cell Imaging System (Essen BioScience). High quality phase-contrast images of 16 fields in each well were taken in 6-hour interval for 4 days (SK-BR-3 and SK250LR) or 7 days (BT-474 and BT250LR). Culture medium and drugs were replaced on day 2 and day 5. The metric of monolayer confluence was measured by IncuCyte software package Confluence v1.5 and used as a surrogate for cell number. The growth rate of cells was calculated by plotting the log of confluence against time. The percentage growth rate is calculated with respect to DMSO control.

### Cell proliferation assay

Approximately 10,000 cells per well (for SK-BR-3 and SK250LR) or 20,000 cells per well (for BT-474 and BT250LR) were seeded in triplicate on 24-well tissue culture plates, allowed to adhere and enter log phrase overnight. Each triplicate was treated with the indicated agents for 3 days. Culture medium was discarded. Cells were washed with PBSA twice and then detached from the well with 500 μl of 0.25% trypsin-EDTA solution. Trypsinised cells were diluted using Coulter Isoton II diluent and counted using a Coulter Z2 particle counter (Beckman Coulter); only the cell population with the size of 13–27 μm was counted.

### Apoptotic assay

Approximately 150,000 cells per well were seeded on 6-well tissue culture plates, allowed to adhere and enter log phrase overnight. Each well was treated with the indicated agents for 2 days. Culture medium was collected. The cells were washed twice with PBSA and the PBSA was retained to collect non-adhered cells. The cells were trypsinised, washed and resuspended with their respective culture medium-PBSA mixture. Along with the non-adhered cells, cells were pelleted and washed with PBSA twice. The washed cell pellet was then resuspended in Annexin V-Alexa Fluor 647 (Life Technologies) and propidium iodide (Sigma-Aldrich) solution, and incubated in the dark at RT for 15 minutes. The stained cells were analysed using a CyAn ADP analyser (Beckman Coulter). AV-Alexa Fluor 647 was measured by excitation at 642 nm and emission detection at 665 nm. Propidium iodide (PI) was measured by excitation at 405 nm and emission detection at 675 nm.

### Xenograft experiment

BalB/c-nude mice were purchased from Charles River. All protocols were carried out with appropriate ethical approval under Home Office regulations and the project license of Dr. Ji-Liang Li (PPL 30/2771) as per previous xenograft experiment [46]. At the age of 5–6 weeks, approximately 5×10^6^ BT-474 cells in 1:1 matrigel/RPMI 1640 medium were injected subcutaneously into the mammary fat pad of the mice under general anaesthesia induced by isoflurane. The diet of the mice was supplement by 5ug/ml β-estradiol and 0.04% (v/v) Baytril in drinking water from 2 days before tumour cell implantation until the end of experiment. The xenograft tumour size was monitored twice a week before treatment. When the tumour size of more than 40% of mice reached 100–150 mm^3^ (length×width×height×0.52), the mice were randomised into 4 treatment groups: a) TWEEN80 (control for lapatinib) and PBSA (control for pertuzumab), b) lapatinib and PBSA, c) TWEEN80 and pertuzumab, d) lapatinib and pertuzumab, and they received treatment for 3 weeks. Lapatinib was administered 5 times per week at 50 mg/kg body weight, half of the standard dose [47, 48], by oral gavage. Pertuzumab was administered once per week at 12mg/kg body weight for the first week and 6mg/kg body weight afterwards by intraperitoneal injection [49, 50]. The mice were sacrified when the geometric mean diameter ([length + width]÷2) of the tumour exceeded 15 mm or animal welfare was compromised. The mice were euthanised by cardiac puncture under isoflurane general anaesthesia and death confirmed by neck dislocation. Residual tumours were removed and were immediately fixed in 4% paraformaldehyde at 4°C for 48 hours and stored in 70% ethanol at 4°C until being processed at the Oxford Centre for Histopathology Research. Paraffin-embedded tumours were sectioned into 4 μm slides.

### Immunohistochemistry staining

The tissue sections were incubated at 60°C for 10 minutes and then were immediately washed twice in histo-clear solution (National Diagnostics) for 5 minutes each. Then they were rehydrated twice with 100% ethanol for another 5 minutes each, followed by 70% ethanol for 5 minutes and finally tap water for 5 minutes. The rehydrated tissue sections were put in a vessel containing 1× Target retrieval solution (Dako) or EDTA pH8.0 buffer (according to antibody manufacturer's protocol). The vessel was heated to 125°C for 2 minutes in a pressure cooker. Once the cooker de-pressurised, the tissue sections were rinsed in water and kept in PBS until blocking. The samples were blocked with normal horse serum (available from ImmPRESS kit, Vector Laboratories) at RT for 30 minutes. Primary antibodies diluted in normal horse serum were applied to the slides and incubated at 4°C overnight. The tissue sections were washed in PBS for 5 minutes. Mouse or rabbit secondary antibodies (available from ImmPRESS kit, Vector Laboratories) were applied and incubated at RT for 30 minutes. The tissue sections were washed in PBS for 5 minutes. ImmPACT DAB peroxidase substrate (Vector Laboratories) was diluted according to the manufacturer's protocol and applied to the slides for 1 to 10 minutes (depending on the antibody used), then washed with tap water. Haematoxylin (Sigma-Aldrich) counterstain was applied to the tissue sections for 30 seconds and then washed with tap water. Finally, the tissue sections were mounted with Aquatex agent (VWR).

Primary antibodies for EGFR (1:50), phospho-HER2 (Y1221/22) (1:320), HER2 (1:400) and phospho-HER3 (1:1600) were obtained from Cell Signaling Technology. Phospho-EGFR antibody (1:50) was obtained from Santa Cruz Biotechnology. HER3 antibody (1:1000) was obtained from Abcam. Phospho-HER4 antibody (1:100) was obtained from Novus Biologicals. HER4 antibody (1:200) was obtained from Thermo Scientific. Scoring of IHC staining was performed independently by two authors, WL and IR, based on the intensity of staining and the percentage of cells stained with a particular intensity. The score of each intensity band was calculated by multiplying the intensity score with the percentage of cells in such intensity (e.g. if 20% of cells have 3+ intensity, the score of would be 60). The total score of a sample is the sum of the scores of individual intensity bands; for example, a tumour sample, which has 60% of tumour cell with strong (3+) intensity, 20% of cells with moderate (2+) intensity and 20% of cells with weak (1+) intensity, has a total score of 3×60 + 2×20 + 1×20 = 240. The maximum score of a sample is 300 and the minimum is 0.

### Statistical analysis

Statistical analysis was performed using GraphPad Prism 6. For experiments involving multiple treatment groups and technical replicates (e.g. total cell counting and ELISA), the data were analysed using matched two-way ANOVA with Bonferroni post-hoc correction. For experiments involving multiple treatments without technical replicates (e.g. western blot densitometry and FACS), the data were analysed using matched one-way ANOVA with Bonferroni post-hoc correction. To compare dose-dependent effect on different groups (e.g. lapatinib-sensitive and -resistant cell lines), the parameters of the best-fit line/curve were compared using extra sum-of-squares F test. Comparison of two groups (regression vs. non-regression) was performed using unpaired t-test.

## SUPPLEMENTARY MATERIAL FIGURES



## Abbreviations

NRG1: neuregulin-1
HER (ErbB): human epidermal growth factor receptor
mAbs: monoclonal antibodies
TKIs: tyrosine kinase inhibitors
T-DM1: Trastuzumab emtansine
pCR: pathological complete response
FDA: Food and Drug Administration
TKD: tyrosine kinase domain
Akt: Protein Kinase B (PKB)
MAPK: Mitogen-activated protein kinase
PFS: Progression-free survival
FOXO3a: Forkhead box O3a
ER: oestrogen receptor
PI3K: Phosphatidylinositol-4,5-bisphosphate 3-kinase
RelA: v-rel avian reticuloendotheliosis viral oncogene homolog A
PBSA: Phosphate buffered saline
RPMI: Roswell Park Memorial Institute medium
EDTA: Ethylenediaminetetraacetic acid
IHC: immunohistochemistry
mRNA: Messenger RNA
SK250LR: SK-BR-3 long-term lapatinib-resistant cells
BT250LR: BT-474 long-term lapatinib-resistant cells
ELISA: Enzyme-linked immunosorbent assay
IgG: immunoglobulin
DMSO: Dimethyl sulfoxide
WD: withdrawal
siRNA: Small interfering RNA
AV: annexin V
PI: propidium iodide
ADAM: A Disintegrin And Metalloproteinase
ADCC: antibody-dependent cell-mediated cytotoxicity
RT: room temperature
