# Supplementary material for: Combining lapatinib and pertuzumab to overcome lapatinib resistance due to NRG1-mediated signalling in HER2-amplified breast cancer
Source: Oncotarget. 2015 Jan 21;6(8):5678–94. doi: 10.18632/oncotarget.3296 (PMC4467394; doi:10.18632/oncotarget.3296)
Supplement: Supplementary file 1 [file oncotarget-06-5678-s001.pdf]

Combining lapatinib and pertuzumab to overcome lapatinib resistance due to NRG1-mediated signaling in HER2 amplified breast cancer

Supplementary Material

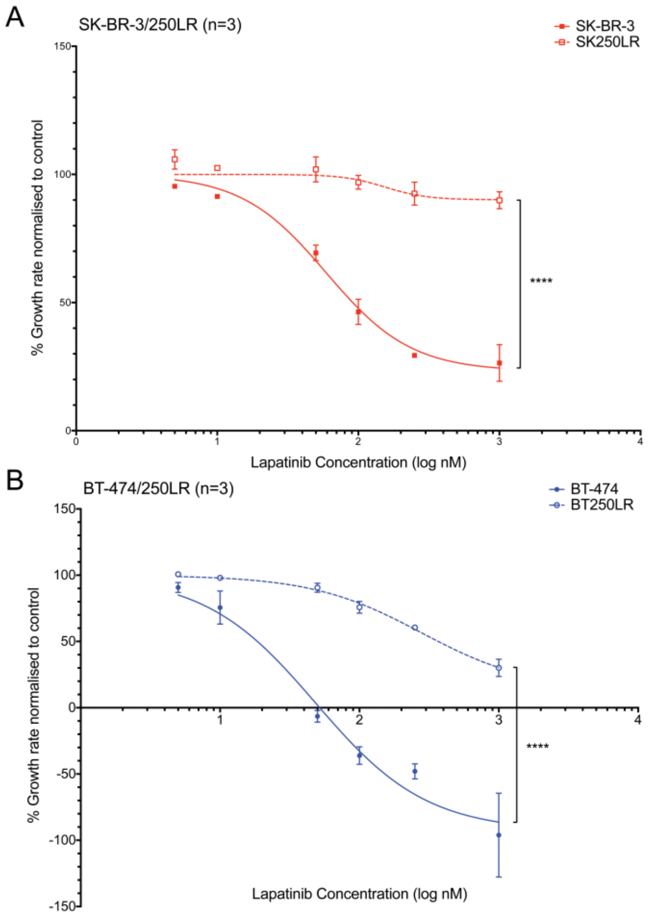

Suppl. Figure 1: Comparison of lapatinib sensitivity between lapatinib-sensitive and -resistant cell lines

(A) SK-BR-3 and SK250LR, (B) BT-474 and BT250LR cell lines were treated with the indicated doses of lapatinib. The growth of cells was monitored using IncuCyte real-time imaging system over 4 days (A) or 7 days (B), with 16 views captured in each well at least every 6 hours. The drug and culture medium were replaced on day 2 and day 5. Three independent experiments were done with three technical replicates, and the confluency of the wells was calculated using manufacturer's algorithm Confluence v1.5. The log of confluency of cells was plotted against time, and the

slope of their linear regression was taken as the magnitude of growth rate. The percentage growth rate with respect to DMSO control was plotted against the log of lapatinib concentration as above. A negative growth rate represents the cells were killed by the treatments. (Error = SD; ****p<0.0001)

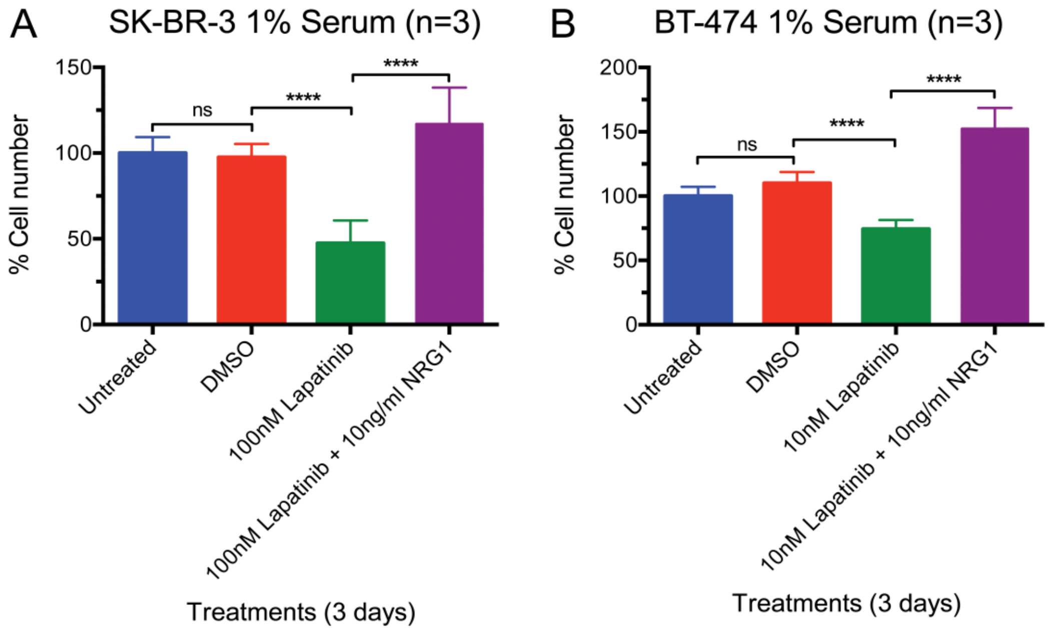

Suppl. Figure 2: NRG1 rescued lapatinib-induced cell inhibition

(A) SK-BR-3 and (B) BT-474 cells were treated with lapatinib or DMSO, with or without NGR1 stimulation for three days before being trypsinised and counted. The percentage cell number was normalised to the untreated/no drug control. At least three independent experiments were done with three technical replicates. (Error = SD; **p<0.01, ***p<0.001, ****p<0.0001)

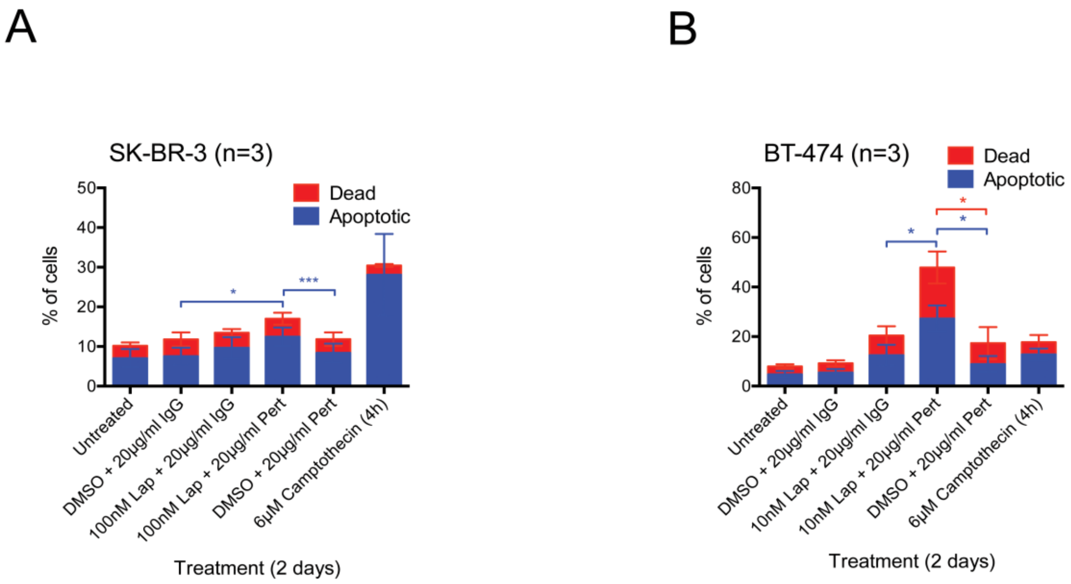

Suppl. Figure 3: The effect of lapatinib and/or pertuzumab on apoptosis in SK-BR-3 and BT-474 cells

(A) SK-BR-3 and (B) BT-474 cells were treated with the indicated treatment for two days. Cells were then trypsinised, collected and stained with Annexin V-Alexa Fluor 647 and propidium iodide. Stained cells were analysed using CyAn FACS analyser. Three independent experiments were performed. (Error = SD; *p<0.05, ***p<0.001, ****p<0.0001)

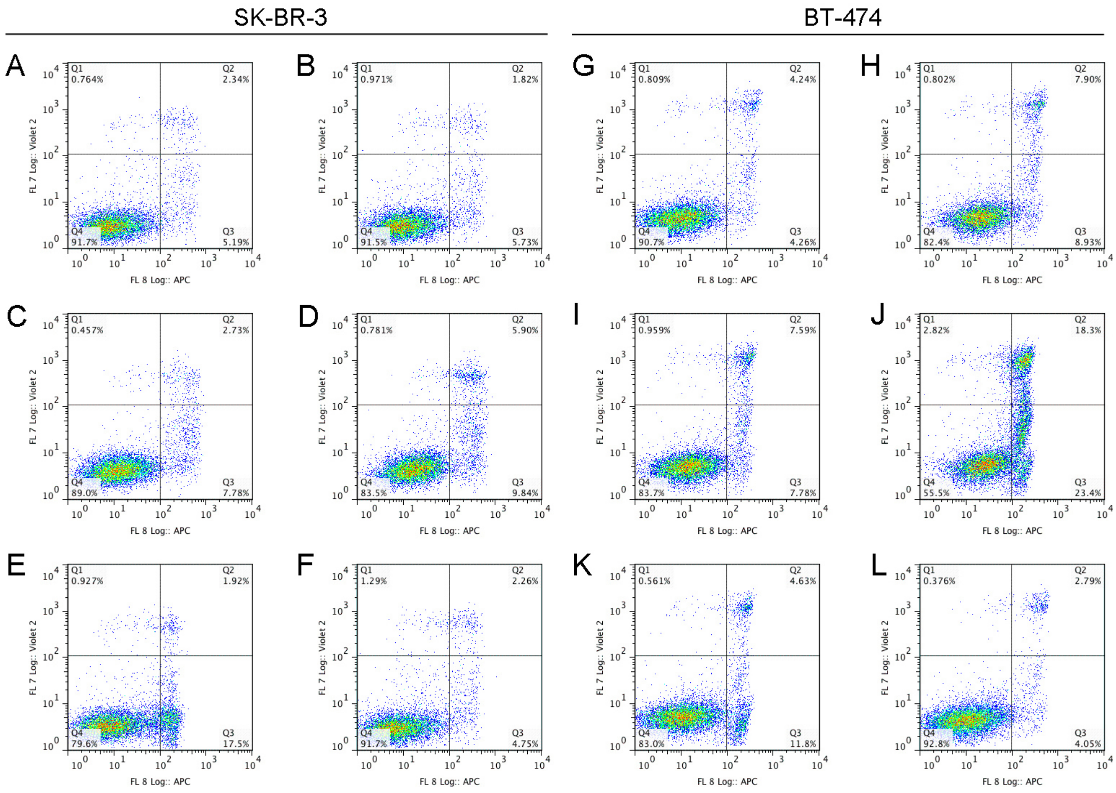

Suppl. Figure 4: Apoptosis analysis of SK-BR-3 and BT-474 cell lines after lapatinib and/or pertuzumab treatment

SK-BR-3 cells were treated with (A) DMSO and 20 μg/ml non-specific human IgG, (B) DMSO and 20 μg/ml pertuzumab, (C) 100 nM lapatinib and 20 μg/ml non-specific human IgG, (D) 100 nM lapatinib and 20 μg/ml pertuzumab, (E) 6 μM camptothecin and (F) no reagent for two days. BT-474 cells were treated with (G) DMSO and 20 μg/ml non-specific human IgG, (H) DMSO and 20 μg/ml pertuzumab, (I) 10 nM lapatinib and 20 μg/ml non-specific human IgG, (J) 10 nM lapatinib and 20 μg/ml pertuzumab, (K) 6 μM camptothecin and (L) no reagent for two days. Cells were trypsinised, collected and stained with Annexin V-Alexa Fluor 647 and propidium iodide. Stained cells were analysed using CyAn FACS analyser. Plots are representative of three independent experiments.

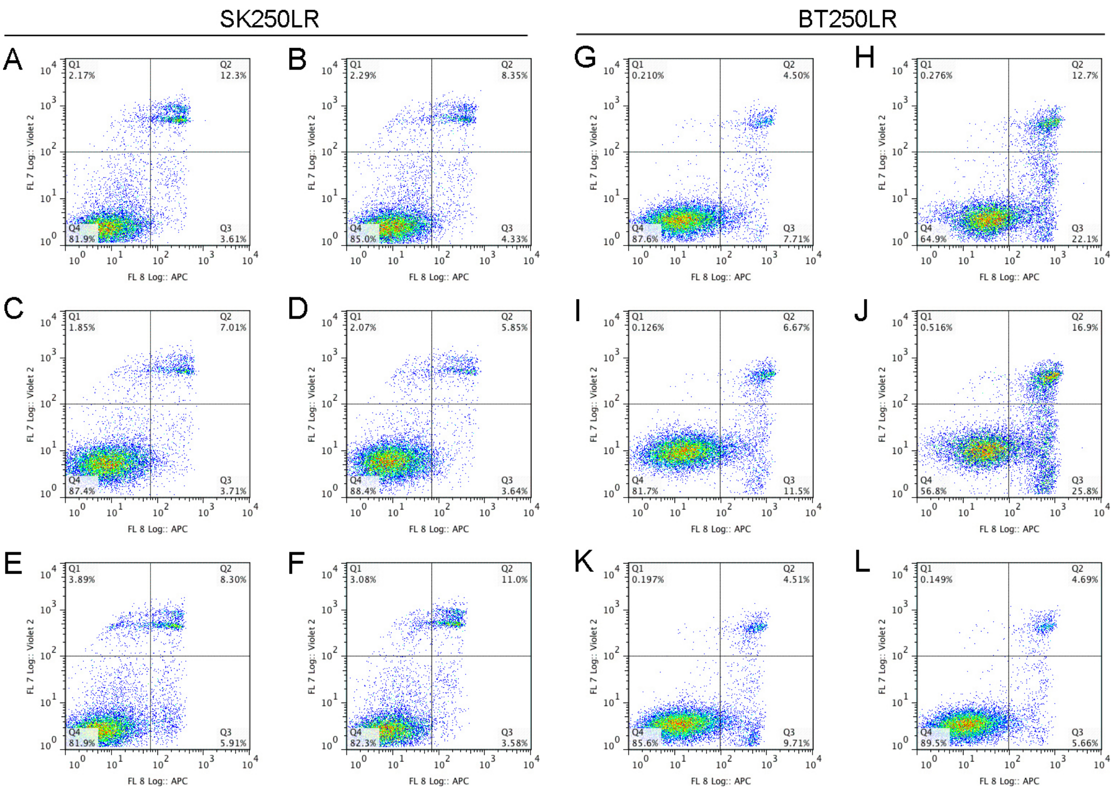

Suppl. Figure 5: Apoptosis analysis of SK250LR and BT250LR cell lines after lapatinib and/or pertuzumab treatment

SK250LR cells were treated with (A) DMSO and 20 µg/ml non-specific human IgG, (B) DMSO and 20 µg/ml pertuzumab, (C) 250 nM lapatinib and 20 µg/ml non-specific human IgG, (D) 250 nM lapatinib and 20 µg/ml pertuzumab, (E) 6 µM camptothecin and (F) no reagent for two days. BT250LR cells were treated with (G) DMSO and 20 µg/ml non-specific human IgG, (H) DMSO and 20 µg/ml pertuzumab, (I) 250 nM lapatinib and 20 µg/ml non-specific human IgG, (J) 250 nM lapatinib and 20 µg/ml pertuzumab, (K) 6 µM camptothecin and (L) no reagent for two days. Cells were trypsinised, collected and stained with Annexin V-Alexa Fluor 647 and propidium iodide. Stained cells were analysed using CyAn FACS analyser. Plots are representative of three independent experiments.

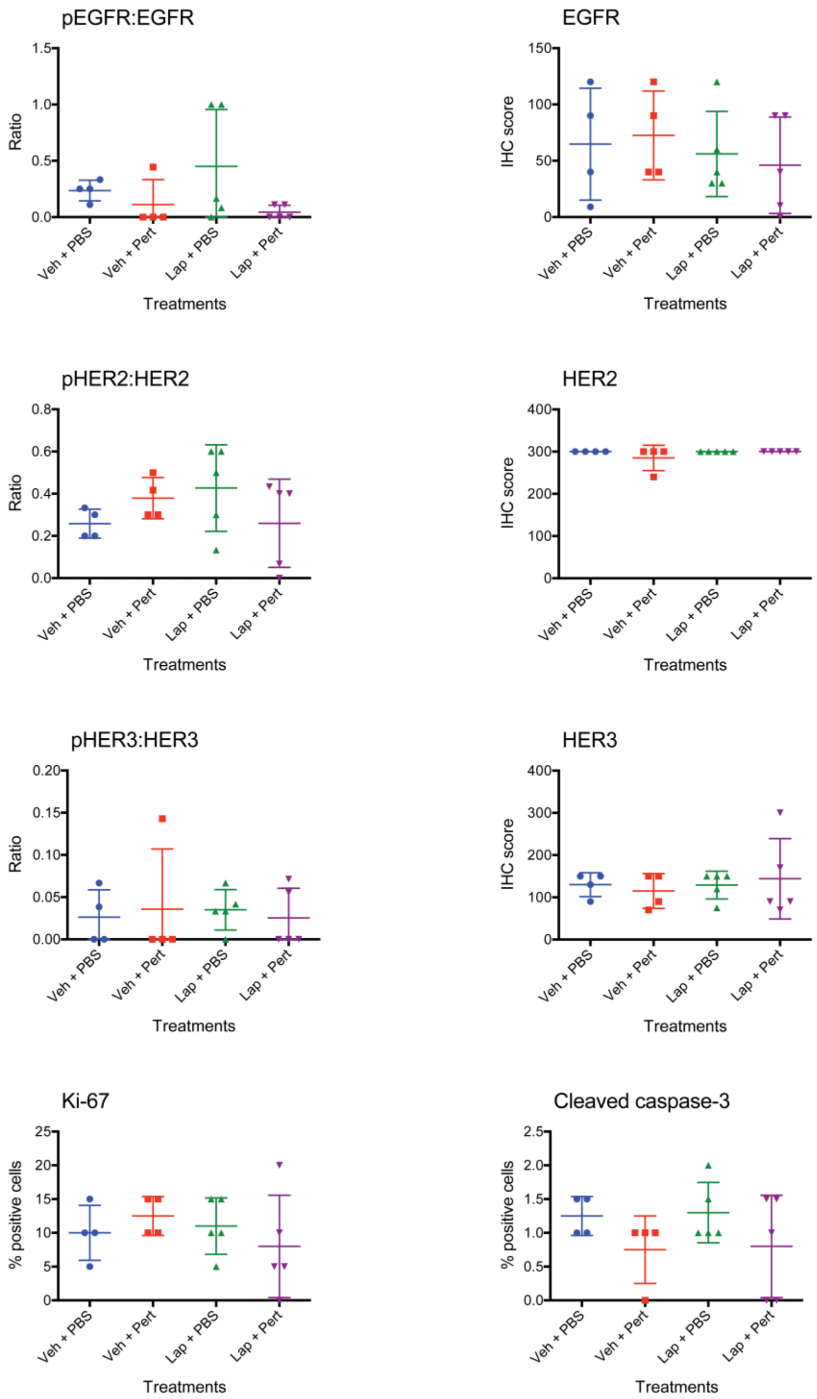

Suppl. Figure 6: Effect of lapatinib and/or pertuzumab treatment on HER signalling, proliferation, apoptosis and necrosis in vivo

Mice were treated with four groups of treatment as described in Figure 4. Mouse xenograft tumours were sectioned into 4 µm slides. IHC staining was performed to the indicated proteins. HER receptors and their phosphorylated forms were scored in terms of staining intensity and percentage of cells stained with the respective

intensity. Ki-67 and cleaved caspase-3 were scored in terms of percentage of positive stained cells. Necrosis was scored in terms of the percentage of necrotic region in a tumour. The score of phospho-proteins was shown in the ratio of phospho:total protein. The scores were then grouped according to the treatments that the mice received. All scorings were performed individually by the author and a pathologist. (Error = SD)

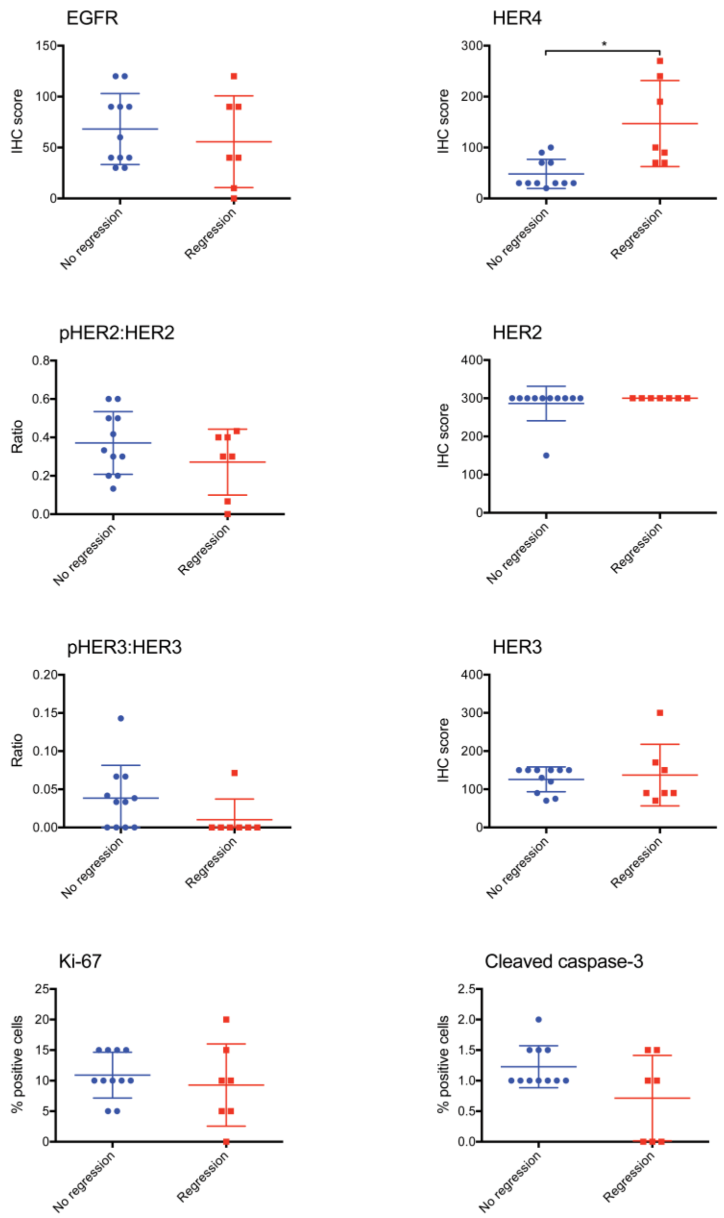

Suppl. Figure 7: Comparison of IHC markers and the regression status of tumours

The IHC scores in Supp. Fig. 6 were grouped according to whether the tumour regressed or not. (Error = SD; *p<0.05)
